# Multi-Tasking Policy Coordination and Corporate Environmental Performance: Evidence from China

**DOI:** 10.3390/ijerph20020923

**Published:** 2023-01-04

**Authors:** Hongji Xie, Cunzhi Tian, Fangying Pang

**Affiliations:** 1 College of Economics, Jinan University, Guangzhou 510632, China; 2 School of Business, Guangxi University, Nanning 530004, China

**Keywords:** economic growth target pressure, environmental protection targets, pollution emission, environmental regulation, Q56, H70, H77

## Abstract

This paper investigates how local governments coordinate the relationship between economic growth targets (EGT) and environmental protection targets (EPT) and the impact of such coordination on firm’s environmental performance. Using the pollution emission data of China’s industrial firms covering 2003 to 2013, we show that firms in the cities where officials are setting overweighted economic growth targets have more sulfur dioxide intensity, while the central government’s hard constraints on EPT included in the official performance evaluation system could partially mitigate the environmental externality of the economic growth target. Further, we find that overweighted EGT significantly decreases firms’ desulfurization facilities, capacity, and ratio, while the hard constraint of EPT helps mitigate this negative relationship. We also find that the positive relationship between overweight EGT and firm emissions is more pronounced in the dirty industry, while the hard constraint of EPT helps to mitigate this relationship. The above results help to identify an underlying mechanism of environmental regulation. Finally, we show that converting the hard constraints of environmental protection targets to self-constraint by local government officials could reverse the environmental externality of the economic growth target.

## 1. Introduction

In the past, an undue focus on GDP growth targets allowed Chinese governments to claim economic victories while ignoring sustainable development. However, as China has clearly put forward the policy targets of “*carbon peak by 2030*” and “*carbon neutral by 2060*”, government officials face higher requirements for coordinating the relationship between economic development and environmental sustainability. On the one hand, the local government can no longer unilaterally pursue high-speed economic growth at the expense of environmental pollution. On the other hand, they cannot formulate carbon reduction policies that are too ambitious and unrealistic and that damage the economy’s long-term growth potential. In this study, we examine how local governments coordinate the policy targets of economic development and environmental protection based on the institutional background of China, as well as the resulting environmental governance performance.

Our research is motivated by China’s unique system of policy targets, which helps to better identify the economic policy pressures facing local governments and how they balance multiple policy targets. Against the backdrop of China’s “economic-first” policy and sole-party political system, the economic growth targets formulated by Chinese governments at all levels are closely related to the assessment and promotion of local officials [1,2]. Therefore, local governments reaching or exceeding the economic growth targets would largely outnumber the governments that fail targets [1]. Lower-level officials are generally placed in a tournament-based promotion system through a combination of political incentives and economic-growth-target pressure [3,4,5]. When officials are intent on meeting or exceeding economic growth targets, they can potentially relax local environmental regulations, which in turn may enable them to pursue excessive capital investment and rapid economic growth at the cost of excessive energy consumption and environmental quality deterioration [6,7]. Such variations could capture local officials’ different economic growth target pressures. Thus, we can better identify how local government officials make trade-offs on multi-tasking policy targets under pressure and how to influence firms’ pollution behavior.

In addition, market participants face unique institutional environments in transition economies, such as government intervention, frequent policy adjustments, inadequate property rights protection, imperfect market mechanisms, and political connections [8]. Such policy interventions could have far-reaching implications for market micro-entities. In particular, the performance evaluation system for local officials has been constantly adjusted over the past ten years (For example, the “Eleventh Five-Year Plan” promulgated in 2006 clearly defined “10% reduction in the total discharge of major pollutants” as a binding target. The “Decision on Implementing the Scientific Outlook on Development and Further Strengthening Environmental Protection” issued by the State Council in December 2006 clearly stated that the pollution reduction performance of cadres should be used as the basis for the performance evaluation system. In 2007, the “Measures for the Assessment of Total Emission Reduction of Major Pollutants” explicitly mentioned the “one-vote veto” system. In 2013, the Organization Department of the CPC Central Committee issued the “Notice on Improving the Performance Assessment of Local Party and Government Leading Groups and Leading Cadres”, which made structural adjustments to the performance evaluation system for local officials, clearly proposed to weaken the assessment weight of GDP growth and strengthen constraints such as environmental protection targets), strengthening the incentive and assessment of environmental protection targets. Will this policy adjustment help alleviate the short-sighted behavior of local officials and coordinate multi-tasking policy targets in environmental governance performance?

Using panel data of 105,339 industrial firms and 322,212 observations from 2003–2013, we find that firms in cities where officials are setting overweighted economic growth targets release more SO_2_ than other firms. The conclusion remains robust after using the instrumental variable model and a series of robustness tests. We identify an underlying mechanism of the relaxation of environmental regulation through which economic-growth-target pressure exacerbates pollution emission. Further, we investigate how environmental protection targets’ hard constraints affect the environmental externalities of economic growth targeting based on the central government’s hard constraints on environmental protection targets included in the official performance evaluation system in 2006. Finally, we explore whether local government officials can reverse the trend of environmental externalities of economic growth targets by turning external hard constraints of environmental protection targets into an internal self-constraint.

Our research makes three marginal contributions. First, this paper expands the existing literature’s understanding of the relationship between economic development and environmental protection from the perspective of policy targets. We explore how local governments balance the relationship between economic growth and environmental protection from a new perspective of policy targets and conduct heterogeneity analysis from the characteristics of policy targets. In addition, our empirical strategy ensures not only the direction of environmental performance of the multitasking policy targets but also the magnitude of the effect.

Second, our research expands our knowledge of corporate environmental performance at the macro-policy level. Prior research made analyses using factors at the capital market or the firm level [9,10]. Instead, we discuss the determinants of firm-level pollution emissions from the macro level, enriching research on firm pollution emissions. This study is also helpful to supplement research dedicated to exploring how macro policies affect micro firm ESG performance.

Third, the extant literature on the economic effects of economic growth targets has focused on economic growth [5], total factor productivity [11], investment activities [2], and innovation [12], but they lack insights into the external outcomes of non-economic activities, especially of corporate environmental behaviour. This study confirms that economic growth target pressure has environmental externalities and complements prior research on the economic effects of economic growth targets.

## 2. Research Hypotheses

Performance targets are widely used to incentivize managers in modern organizations [13]. In essence, whether targets can lead to better performance depends on the underlying incentive mechanism and target constraints. According to the multitask principal-agent model, an imbalance in an agent’s compensation for any task would cause the reallocation of attention away from other tasks [14]. When agents deal with various tasks and have limited resources, they will prioritize tasks that are more important, that are easier to measure, and that take less time for completion.

Local governments in China are often required to assess multiple indicators, including economic growth, people’s livelihood expenditure, and environmental protection. Compared with the assessment procedures for environmental protection (which are rather complex), those for economic growth are simpler, more direct, and more efficient (e.g., direct GDP measurement). It is not hard to rationalize that local officials will prioritize economic growth targets over the hard-to-assess sustainability targets. In addition, the opportunity cost for local governments to pursue environmental protection targets is rather high, which may intensify the prioritization of growth targets.

To understand the importance of economic growth goals to the utility function of local officials, we should look at the top-down political incentive and official promotion system. Vertical management across different levels of government administration and the evolution of promotion tournaments have led to a top-down target incentive system, motivating officials to set high local targets and exceed overweight GDP targets [15,16]. Local officials are internally motivated to seek political career promotion, and meeting economic growth targets has become an important means for lower-level officials to transmit signals to their superiors [2]. Research shows that setting and exceeding high economic growth targets is crucial for lower-level officials to prove their governance capability [17]. In addition, lower-level governments often regard the targets set by higher-level governments as a bottom line when formulating their plans for the future, leading to a target-setting competition among governments at different levels.

When the political performance evaluations of lower-level officials conducted by higher-level governments are based heavily on GDP growth, local officials may focus and choose to act toward accomplishing the related tasks set by the superior government [18]. Then, since economic growth is the core criterion for promotion evaluation, the pressure on local officials surges significantly if the actual growth rate is lower than the target [2]. This situation may lead lower-level officials to resort to strategies that yield only short-term outcomes but that will also ensure that they meet the set targets [1,2].

Against the background of the aforementioned promotion tournaments, when intent on meeting or exceeding economic growth targets, local governments can relax local environmental regulations, which in turn enables them to pursue excessive capital investment and rapid economic growth at the cost of excessive energy consumption and environmental quality deterioration [6,7]. It occurs because energy- and pollution-intensive industries can provide a rather significant economic output in a relatively short period, so local officials are often inclined to implement policies that benefit these industries [19].

From the perspective of productive firms, following rigid environmental protection regulations requires an increase in pollution-control expenditure, adding to the total productive costs [10,20]. The existing literature shows that earnings pressure from the capital market increases SO_2_ emission intensity by 26.7% and that the cost savings from the additional emissions reduce earnings per share by 0.4–1.4 cents [10]. Further, in the past few decades, environmental fines in China have been lower than the cost of emissions reduction (The existing literature documents that the pollution cost of SO_2_ in 2005 was 0.63 CNY/kg, while the emission reduction cost was 4–6 CNY/kg [17]), implying that firms have little incentive to reduce their emissions. In practice, firms can also bypass pollution-control measures by manipulating the operating frequency of the SO_2_ scrubber. For instance, firms can operate the SO_2_ scrubber only upon the imminence of inspection to reduce costs and still pass the inspection without any problems [21,22]. Together, all these delineations lead to hypothesis H1.

**H1.**  *Ceteris paribus, cities with greater economic-growth-target pressure will show higher SO_2_ emission intensity at the firm level*.

To solve the dilemma of inconsistent policy targets, the central government has continuously adjusted the performance evaluation system for local officials since 2007, including weakening the weight of GDP indicators, clarifying the specific emission reduction values of specific pollutants for emission reduction targets, and decomposing them to the provincial and municipal levels. In addition, emission reduction targets are also linked to official promotion evaluation and even set up a “one-vote veto”. This means that environmental protection targets change from soft to hard constraints [23]. The adjustment of the performance evaluation system changes the promotion incentives faced by local officials, directly affects their utility functions and strategic responses, drives local officials to trade off the relationship between economic development and environmental protection targets [24], and gives a higher priority to achieving environmental policy targets. Therefore, we propose hypothesis H2:

**H2.**  *Ceteris paribus, the hard constraints of environmental protection targets help to alleviate the positive effects of economic-growth-target pressure and firm emission*.

## 3. Research Design

### 3.1. Data Sources and Sample

Considering that industrial firms are the primary source of pollution emissions [10], we choose industrial firm-level data to measure pollution emissions at the micro level. We collect more than 300,000 industrial firms’ emission data from 1998–2014. The data comes from the China Environmental Survey and Reporting Database (ESR) obtained from the Ministry of Environmental Protection of China, which contains rich information on firms’ environmental behavior and reliable data quality [10,25]. We use the China Industrial Enterprise Database (CIED) to capture basic information, geographic location, ownership, and the financial characteristics of firms. Following the existing literature [26], we conduct explicit one-to-one matching according to identical information (e.g., legal person codes and firm full names) from the CIED and ESR datasets. Furthermore, we manually collect data on GDP growth and environmental protection targets from both provincial and prefectural governments across China, starting from 2002. We collect these data from two sources: the governmental websites, statistical yearbook, or Internet search. Other municipal and provincial statistics were obtained from the statistical yearbook of the local government.

The sample period covers a decade from 2003 to 2013 because the CIED was updated until 2013. In total, we obtain a panel sample of 322,212 observations covering 105,339 industrial firms. In addition, we follow the existing literature on data-cleaning procedures [26], eliminating firms with: (1) missing data on major financial indicators (e.g., total assets, net fixed assets, number of employees, and total industrial output value); (2) fewer than ten employees; (3) financial indicators that are inconsistent with generally accepted accounting principles or GAAP (e.g., total assets less than net assets or with a profit margin of more than one); and (4) pollutant emissions less than zero; and (5) observations from 2010 with serious data quality problems. All continuous variables are winsorized at the 1st and 99th percentiles to mitigate the effect of outliers.

### 3.2. Main Variable Definition

#### 3.2.1. Economic-Growth-Target Pressure

**Figure 1:**
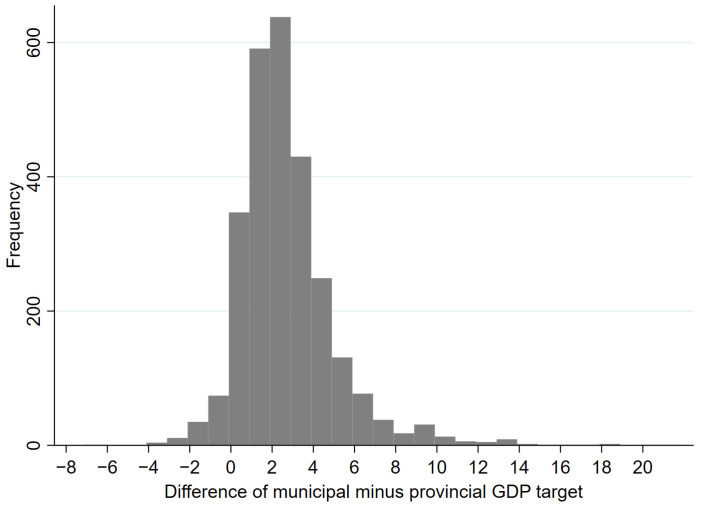
The distribution of the difference between the municipal and provincial GDP targets.

Due to economic development differences among regions, there are endogenous variances in formulating their respective economic growth targets. For example, developed regions have better economic performance and have already gone through a period of rapid economic growth, so their economic growth targets are lower than those of developing regions. Therefore, the absolute value of the economic-growth-target rate does not suffice to outline lower-level governments’ target pressure or motivation. In the political promotion tournament, lower-level officials who aspire to be promoted send positive signals and demonstrate their competitiveness to higher-level governments by establishing targets and tasks that correspond to the growth targets set by the higher-level government. Owing to this reality, the phenomenon of “top-down amplification” often occurs in economic growth targets set across different administrative levels of the Chinese government [5]. Figure 1 reports the distribution of the difference between the municipal and provincial GDP targets. This top-down amplification is essentially the “self-constraint” of lower-level officials in setting economic growth targets. It means that lower-level governments set up overweighted targets; then, they often engage in short-term economic behavior to meet these overweighted growth targets.

Accordingly, we measure the economic-growth-targets pressure on local officials based on whether the economic growth targets set by municipal governments exceed those set at the provincial level. Specifically, we construct the dummy variable EGT, which takes the value of one if the GDP growth target set by the municipal government in a given year is higher than the provincial GDP growth target and 0 otherwise.

#### 3.2.2. Hard Constraints of Environmental Protection Target

The national “Tenth Five-Year Plan”, promulgated in 2001, specified a target of 10% emission reduction of major pollutants (SO_2_ and COD) at the national level for the first time. However, this expected target was not linked to the performance evaluation system for local officials, which means that the planned emission reduction target lacks a strong constraint. As a result, by the end of 2005, the emission reduction targets of the two major pollutants stipulated in the “Tenth Five-Year Plan” had not been achieved. In sharp contrast, the national “Eleventh Five-Year Plan” issued in 2006 proposed that the primary national pollutants’ binding indicators needed to be reduced in total emissions by 10% at the end of the eleventh five-year plan. Subsequently, the Ministry of Environmental Protection signed the responsibility letter for the total reduction of major pollutants with all provincial governments, emphasizing that “binding indicators” have been included in the performance evaluation system for local officials and used as an important indicator for political promotion. This marks the official transition of China’s environmental protection goals from “soft constraints” to “hard constraints” [23]. Based on the above background, we construct an environmental policy objective hard constraint variable (EPThc), assigning a value of one to the sample observations from 2006 to 2013, indicating that environmental policy targets rigidly constrain local officials from their superiors; the years before 2006 are assigned a value of zero, indicating that local officials face soft constraints on environmental policy targets.

#### 3.2.3. Firm-Level Pollutant Emission Intensity

Following the existing literature [10], we select SO_2_ emissions as a proxy for environmental pollution emissions at the firm level. During our sample period, SO_2_ emissions were one of China’s primary sources of air pollution. Therefore, SO_2_ control and enforcement have been the focus of air-pollution efforts in China. According to China’s five-year development plans, SO_2_ was one of the most critical energy conservation and emission reduction indicators in 2001–2015, and there were specific emission reduction targets for SO_2_. In addition, compared with other pollutants, SO_2_ emissions are a major concern in environmental law enforcement departments, and the related data are released with accuracy and reliability [10]. Owing to SO_2_ emission disparities, we scale the total SO_2_ emission by the total output adjusted for inflation (which was measured by the consumer price index), which we then use as a proxy for pollution emission intensity (The SO_2_ emission data are measured in kgs, and the unit value of output is 1000 CNY. We convert the values of total output produced into constant 2013 prices using the consumer price index for industrial products. We obtained the consumer price index data from the National Bureau of Statistics of China).

### 3.3. Estimation Framework

To test hypothesis H1, we use the pooled OLS model to investigate the relationship between economic-growth-targets pressure and firm-level pollution emission. Specifically, we construct the following regression Equation (1):

Emission_i,t_ = β_0_ + β_1_EGT_c,t_ + β_j_Control_i,t_ + Year FE + Industry FE + City FE + ε_i,t_ (1)

where subscripts i, c, and t represent the firm, city, and year, respectively. Emission_i,t_ is the SO_2_ emission intensity of firm i in year t. EGT_c,t_ indicates whether the GDP growth target set of city c in year t exceeds the provincial target. We suppose that cities with overweighted set of economic growth targets have higher firm-level pollution emission intensity; namely, coefficient β_1_ is significantly positive. Year FE, Industry FE, City FE and ε_i,t_ denote year fixed effects, industry fixed effects, city fixed effects, and random error term, respectively. We control a set of control variables that may affect firm-level pollution emission intensity at firm, municipal, and provincial levels.

To test hypothesis H2, we add the interaction term of economic-growth-targets pressure (EGT) and the environmental policy target (EPThc) based on Model (1), and construct the following regression Equation (2) (Since we control the year fixed effect, EPThc is perfectly collinear with the year dummy variable, so it is omitted and not included in the equation):

Emission_i,t_ = β_0_ + β_1_EGT_c,t_ +β_2_EGT_c,t_ × EPThc_c,t_ + β_k_Control_i,t_ + Year FE + Industry FE + City FE + ε_i,t_ (2)

where subscripts i, c, and t represent the firm, city, and year, respectively. EPThc_c,t_ indicates that city c faces hard constraints on environmental policy targets in year t. We expect the coefficient β_2_ of interaction term (EGT_c,t_ × EPThc_c,t_) to be negative. Other settings of the Model (2) are the same as Model (1).

Following the existing literature [10,25,27], we control the following variables in Model (1) and Model (2): firm size (SIZE), profitability (return on assets; ROA), leverage (Lev), firm age (AGE), the property rights of firms (SOE), per capita GDP (PERGDP), environmental regulation (ERS), and provincial-level marketization degree (MKTIDX). Detailed definitions of key variables are presented in the Appendix A.

### 3.4. Summary Statistics

#### 3.4.1. Sample Composition

Panel A of Table 1 shows the sample distribution by year. We scale the raw amount of SO_2_ emission measured in kgs by the total inflation-adjusted value of output; the average emission represents the SO_2_ emission intensity per thousand CNY of output. For the whole sample, the mean Emission is 0.79, so an average firm in our sample releases 790 g of SO_2_ per thousand CNY of output. The average pollution emission of SO_2_ shows a downward trend, decreasing by half nearly twice in the sample period (from 1.53–0.53) and reflecting China’s increasingly stringent environmental regulations over the past two decades. In particular, emissions declined after the central government included environmental protection targets in officials’ promotion assessment system in 2006, marking the incorporation of environmental targets into the assessment indicators of officials.

Panel B of Table 1 presents the sample breakdown by industry. The industry with the largest SO_2_ emission was the production and supply of electric and heat power, indicating that products in this industry release 7.98 kg of SO_2_ per thousand CNY of output. According to the BP World Energy Statistical Yearbook, China’s coal-fired power generation—a major emitter of SO_2_ and nitrogen oxide—accounted for 78.2% of China’s total power industry in 2013 (Details are shown from https://www.bp.com/zh_cn/china/home/news/reports.html (accessed on 29 October 2021)), which is consistent with our data. Other industries with emission values larger than one are non-metallic products, paper and allied products, non-metallic mining, gas production and beneficiation, petroleum, and the nuclear fuel industry, all of which have a single commonality: they are high-energy-consuming and -polluting industries.

#### 3.4.2. Descriptive Statistics

Table 2 reports the descriptive statistics of the variables of interest. First, the mean emission is 0.79, meaning that an average firm in our sample releases 790 g of SO_2_ per thousand CNY of output. The standard deviation of Emission in our sample is 2.56, suggesting great differences among firms. Regarding independent variables, the mean value of EGT is 0.84, indicating that 84% of the observations are in municipal governments that set economic growth targets higher than the provincial targets. The mean value of EPThc is 0.767, indicating that 76.7% of the observations are in the period of hard constraints on environmental policy targets, i.e., 2006–2013.

## 4. Policy Targets Coordination and Firm Pollution Emission

### 4.1. Baseline Regression Results

Table 3 reports the results of baseline regression Models (1) and (2). Columns (1)–(2) in Table 3 provide the results of the impact of EGT on pollution emission in all specifications of Model (1). In column (1), we only add control variables of firm characteristics and the fixed effects of year, industry, and city to control for the time-invariant and unobservable industry and city characteristics, and the coefficient of EGT is significantly positive at the 1% level (T value = 2.68). In column (2), we further add the control variables of municipal and provincial characteristics, showing that the coefficient of EGT is also significantly positive at the 1% level (T value = 3.32). In sum, the coefficients of EGT in all specifications are significantly positive, indicating the positive relationship between the top-down amplification of a city’s economic growth target and firm-level pollution emission. Regarding economic significance, SO_2_ emission increases about 48–60 g per thousand CNY of output in the year when the local government overweight the economic growth target; i.e., it increases from 6.11% (0.048/0.786) to 7.63% (0.06/0.786) compared with the average SO_2_ emission (0.786). The results show the economic magnitude of the baseline Model (1). Under China’s economy-first policy and strong promotion incentives and to ensure that the economic growth targets are met or exceeded, local officials would distort environmental regulation implementation, affecting firm behavior and increasing pollution emissions at the firm level. Thus, our results support H1.

Columns (3)–(4) in Table 3 give the regression results of the benchmark regression Model (2) under different sets of control variables. Results show that the coefficient of EGT is still significantly positive at the 1% level under different control variable sets. The economic implication behind it is that SO_2_ emission increases about 151–154 g per thousand CNY of output in the year when the local government overweights the economic growth target; i.e., it increases from 19.21% (0.151/0.786) to 19.59% (0.154/0.786) compared with the average SO_2_ emission (0.786). The results help to understand that China’s SO_2_ emissions during the “Tenth Five-Year Plan” period failed to meet the control target of a 10% reduction but increased significantly by 27.8% compared to 2000 (More details: http://www.gov.cn/zwgk/2007-11/26/content_815498.htm (accessed on 30 November 2021)). The results also suggest that, when local government officials are faced with the trade-off between the soft constraint of environmental protection targets and the realization pressure of the economic growth target, they give priority to the explicit and easy-to-observe economic policy target at the expense of the implicit and non-hard constraints of environmental protection targets.

The coefficient of EGT × EPThc is significantly negative at least at the 1% level, indicating that environmental protection targets’ hard constraints can effectively reduce industrial firm SO_2_ emission intensity by 131–142 g per thousand CNY of output. Compared with the mean SO_2_ emission intensity of firms (0.786), it decreases about 16.67% (−0.131/0.786) to 18.07% (−0.142/0.786). The results suggest that the hard constraints of environmental protection targets by the central government are an essential factor in alleviating the conflict of multi-tasking policy targets and partially alleviating the negative environmental externalities of the economic growth target, supporting H2.

It is worth noting that the sum of the coefficients of EGT and EGT × EPThc is greater than zero and that the SO_2_ emission intensity increases by 9–23 g per thousand CNY of output. This result shows that the external hard constraints of environmental protection targets are not enough to reverse the performance view of local officials giving priority to GDP and the negative environmental externalities of the economic growth target.

### 4.2. IV Estimations

Considering that the relationship between economic-growth-target pressure and firm pollution emission behavior may be endogenous, we construct a desirable instrument variable for economic-growth-target pressure. Following the method proposed by the existing literature [2], we select the proportion of other cities in the same province that overweigh their economic growth targets higher than the province level (EGTiv) as an instrumental variable for economic-growth-target pressure.

Regarding relevance conditions, horizontal economic competition among governments at the same level can affect the actual behavior of cities at the same level [28,29]. As described earlier, lower-level governments tend to set growth targets higher than those set by higher-level governments, which is aimed at ensuring that they win the promotion competition. Therefore, the top-down amplification of a city’s economic growth target is influenced by the top-down amplification of other cities’ economic growth targets at the same level, thus satisfying the correlation condition. Regardingthe exclusivity of instrument variables, the top-down amplification of other cities’ economic growth targets is unlikely to affect firm-level pollution emissions through other channels instead of amplifying target pressure under promotion tournaments. In addition, since the EPThc is determined by the higher-level government, it is exogenous to local officials. Therefore, we generate a new interaction variable, EGTiv × EPThc, to add to Model (2).

Table 4 shows the regression results for the IVs. The first-stage regression results are shown in columns (1) and (3) of Table 4. The instrumental variable EGTiv is significantly and positively correlated with the endogenous variable EGT, indicating that the higher the proportion of the top-down amplification of other cities’ economic growth targets in the same province, the higher the probability of the top-down amplification of a city’s economic growth target. We perform under-identification and weak-identification tests to verify the validity of the instrumental variable in Table 4. The partial F-statistics of the first-stage regressions are significantly larger than the conventional threshold of 10, indicating that the instrumental variables satisfy the correlation condition. Furthermore, the values of Cragg–Donald Wald F-statistics and Kleibergen–Paap F-statistics in the test of weak IVs are greater than the critical value of 16.38, indicating that there is no weak IV selection problem in this study. The second-stage regression in columns (2) and (4) results show that the coefficient of EGT is statistically positive at the 1% level and that the coefficient of EGT × EPThc is statistically negative at the 1% level; i.e., after alleviating the endogeneity problem, the baseline results of Table 3 still hold.

### 4.3. Other Robustness Tests

To assess the sensitivity of our baseline results, we conduct a battery of robustness checks. As a result, the baseline results remained robust.

First, to exclude the concern that the baseline results are influenced by particular pollutants, we include more indicators and re-estimate the baseline specifications. We use the following pollutant emissions and scale each of them by inflation-adjusted industrial output: chemical oxygen demand (COD), industrial waste gas (IWG), and wastewater (IWW). Table 5 reports the results of alternative measures of pollution emission intensity. Most coefficients of EGT are significantly positive at the 1% level, and most coefficients of EGT × EPThc are significantly negative at the 1% level. In sum, regardless of pollutant type, the conclusion is still consistent with baseline results in Table 3.

Second, we consider sample selection under different circumstances to conduct robustness checks. First, we use data from firms with a business income of CNY 20 million and above during the period of 2011–2013 (when the criteria for a firm to be above the designated size in the CIED changed) and exclude the data from firms with a business income of less than CNY 20 million. Second, 28.15% of the firms have zero emissions (Emission is equal to 0), so we delete their observation data and re-estimate the baseline specifications to ensure that these samples did not affect the baseline results. Third, the central government explicitly proposes to weaken the weight of economic growth targets in the assessment system of local officials and strengthen the assessment of environmental protection in 2013. To ensure that this policy shock does not affect the results of this study, we remove the 2013 sample and re-estimate the baseline regression model. The results in Table 6 show that the baseline estimation is not determined by sample selection.

Third, we also conduct the following robustness tests. First, following the existing literature [24], we replace Emission by taking the logarithm of SO_2_ emissions (LnSO_2_) and by using firm operating income adjusted by the price index to standardize the SO_2_ emissions of firms (Emission_sale). Second, we add firm fixed effects to address the endogenous problems caused by unobservable firm heterogeneity and time-invariant omitted variables. Table 7 reports the results of the above cases; the coefficients of EGT are significantly positive at least at the 1% level, and the coefficients of EGT × EPThc are significantly negative at least at the 5% level, consistent with the baseline results in Table 3.

## 5. Underlying Mechanism: Environmental Regulation

In this section, we explore the underlying mechanism by which the coordination of multi-tasking policy targets affects pollution emissions, namely the environmental regulation channel. Although local governments are assessed for various targets simultaneously, meeting or exceeding the economic growth target remains their primary task. When local governments face economic-growth-target pressure, the opportunity cost for pursuing environmental protection is high, prioritizing economic growth targets. Under such circumstances, local governments are more inclined to engage in short-term behavior and are strongly motivated to relax environmental regulation implementation [30]. However, when the higher-level government turns environmental policy targets into hard constraints and links them to the performance evaluation system, the weight of environmental policy targets in the promotion function of officials increases. This shift will drive local government officials to strengthen environmental regulations [24] and require firms to reduce emissions to meet the assessment requirements of higher-level government.

We first verify the environmental regulation channel directly from the firm level to investigate the environmental regulation channel. Since the detailed desulfurization data of some firms are disclosed in the firm emission database, we construct the following three variables to measure the intensity of environmental regulation faced by firms: (1) the number of desulfurization equipment (LnDnum) proxied by the natural logarithm of the number of sets of desulfurization facilities installed in the firm; (2) the hourly desulfurization capacity of the desulfurization facilities (LnDcap), proxied by the natural logarithm of the hourly desulfurization volume (kg) of the desulfurization facilities; (3) the sulfur dioxide removal rate (RemoveRate), proxied by the ratio of sulfur dioxide removal to sulfur dioxide production.

We re-run Models (1) and (2) using the above variables as dependent variables instead of Emission. The results are shown in panel A of Table 8, where the coefficients of EGT are all significantly negative, at least at the 10% level. It indicates that the top-down amplification of a city’s economic growth target significantly decreases firms’ desulfurization facilities, capacity, and ratio, confirming local officials’ tendency to relax the intensity of environmental regulations when the pressure on economic growth targets is high. Furthermore, the coefficient of EGT × EPThc is significantly positive in columns (2), (4), and (6), indicating that the environmental protection target’s hard constraint strengthens firms’ desulfurization facilities, capacity, and ratio. It confirms the potential channel for local governments to strengthen the intensity of environmental regulation under the hard constraint of the environmental protection targets.

In addition, we also verify the environmental regulation channel indirectly from the industry level. Energy- and pollution-intensive industries (namely, dirty industries) can provide a rather significant economic output in a relatively short period. Therefore, local officials are often inclined to implement policies that benefit these industries when they need to meet economic growth targets [19]. If local officials lead to the increase of SO_2_ by relaxing environmental regulations to achieve economic growth, this positive effect is more significant in dirty industries. In addition, when the hard constraints of environmental policy targets drive local governments to strengthen environmental regulations, the moderating effect is also more pronounced in dirty industries.

Following the existing literature’s approach to the division of heavy-pollution industries [24], we use the CIED to identify the number of heavy-polluting firms’ exits and obtain the number of heavy-polluting firms in each city each year. Further, we construct the dummy variable (DirtyExit) and take a value of one for DirtyExit if the number of heavy-polluting firms exiting the city in a given year is higher than the city-level median and zero otherwise. Finally, we classify samples into two groups (high-exit group and low-exit group) according to DirtyExit, and re-estimate Models (1) and (2).

The results of Model (1) are shown in columns (1) and (2) of panel B in Table 8. The coefficient of EGT is significantly positive only in the low-exit group of heavy-polluting firms. However, the magnitude and significance level differ in relation to the two groups of firms at the 1% level, indicating that the positive effect of economic-growth-target pressure on firm emissions is mainly driven by the low exit of heavy-polluting firms. The results of the baseline Model (2) are shown in columns (3) and (4) of panel B in Table 8. The coefficient of EGT × EPThc is significantly negative only in the group with more polluting firms exiting, indicating that the moderating effect of the environmental protection targets’ hard constraint to mitigate the negative environmental externality of the economic growth target is mainly driven by the local government to accelerate the exit of firms in the heavily polluting industries. There is a potential mechanism for local governments to strengthen the intensity of environmental regulations under the hard constraint of environmental protection targets.

## 6. How to Reverse Negative Environmental Externalities of Economic Growth Targets

### 6.1. Self-Constraining Characteristics of Environmental Policy Targets

The baseline results show that the hard constraints of environmental policy targets can only partially alleviate but not reverse the negative environmental externality trend of economic growth targets. In this section, we further explore whether local government officials can reverse the trend of negative environmental externalities of economic growth targets by turning external hard constraints of environmental protection targets into an internal self-constraint. The self-restraint feature of environmental protection targets is reflected in the fact that local officials, under the hard constraints of environmental targets, actively disclose specific numerical targets of major pollutants, which to some extent reflects a kind of self-restraint [31].

We decompose EPThc into environmental-policy-target self-restraint variables (EPTsc) and non-self-restraint variables (EPTnsc) by using whether local officials actively disclose the numerical control targets of major pollutants in government work reports. If local officials voluntarily disclose the emission-reduction numerical targets of major pollutants in the government work report after 2006, the EPTsc takes a value of one and zero otherwise; if the local officials did not disclose numerical targets of major pollutants in the government work report after 2006, EPTnsc takes a value of one and zero otherwise. We construct Model (3) and expect the coefficients of two interaction terms to be significantly negative and $\left|\gamma_{2}\right|$ > $\left|\gamma_{1}\right|$.

(3) $${\mathrm{Emission}}_{i,t}=\gamma_{0}+\gamma_{1}{\mathrm{EGT}}_{c,t}+\gamma_{2}{\mathrm{EGT}}_{c,t}\times {\mathrm{EPTsc}}_{c,t}+\gamma_{3}{\mathrm{EGT}}_{c,t}\times {\mathrm{EPTnsc}}_{c,t}+\gamma_{4}{\mathrm{EPTsc}}_{c,t}++\beta_{j}{\mathrm{Control}}_{i,t}^{j}\;+\mathrm{Year}\;\mathrm{FE}+\mathrm{Industry}\;\mathrm{FE}+\mathrm{City}\;\mathrm{FE}+\varepsilon_{i,t}\;\;\;\;\;\;\;\;\;\;\;$$

Column (1) of Table 9 reports the results of Model (3). The coefficient of EGT is still significantly positive at the 1% level, and the coefficient of EGT × EPTsc is significantly negative at the 1% level. More importantly, the sum of the coefficients of EGT and EGT × EPTsc is less than zero. It indicates that the self-restraint of local government officials’ active disclosure of environmental protection targets has a more significant decreasing effect on SO_2_ emission intensity than the increasing effect of the top-down amplification of the economic growth target. Local officials transform the hard constraints into the self-restraint of environmental policy targets endogenously, contributing to reversing the improper view of political promotion that one-sidedly prioritizes the realization of economic growth targets at the expense of environmental sustainability. In sum, the self-restraint of environmental policy targets by local officials helps reverse the negative environmental externality of economic growth targets and truly realize the coordination of economic growth targets and environmental protection targets.

### 6.2. Explicit Constraint Characteristics of EPT

According to multitask principal-agent theory, when agents are faced with multiple targets, they prioritize the task with a higher degree of explicitness [14,32,33]. Based on this theoretical framework, the changes in the dominant degree of the task will drive agents to reallocate their efforts across tasks to cater to the assessment requirements of the principal. Suppose that environmental protection targets have more specific and explicit characteristics. In that case, local officials will make more efforts to promote environmental protection targets by developing and implementing policies targeting SO_2_ emission reduction to achieve the emission reduction targets set by higher-level governments [24]. Therefore, we further examine how differences in the explicit characteristics of environmental protection targets affect the environmental governance effects of policy targets coordination.

Based on the differences in the descriptions of environmental protection targets in local government work reports, we capture the explicit characteristics of environmental protection targets and classify local officials’ self-constraint in environmental protection targets into different levels of explicitness in two dimensions: first, the “pollutant” dimension, i.e., the specific extent to which environmental protection targets mention pollutants in government work reports; second, the “value” dimension, i.e., the specific extent to which environmental protection targets mention numerical control indicators of pollutants in government work reports.

Specifically, we first decompose EPThc into four variables according to the specificity of the pollutant mentioned: explicit mention of the specific pollutant (SO_2_ or nitrogen oxides) control target in the environmental protection target (EPTp1), mention of the energy consumption per unit of GDP control target only (EPTp2), mention of the completion of the higher-level government target only (EPTp3), and facing hard constraints from the higher government but not actively mentioning the environmental protection target (EPTp4). We then replace EPThc with the above four variables and put them into Model (2) simultaneously, along with the interaction terms of the four variables with EGT. The results are shown in column (2) of Table 9. The coefficient of EGT × EPTp1 is significantly negative at the 1% level and outperforms the last three groups of interaction terms regardless of the absolute value of the coefficient and statistical significance. Furthermore, the coefficients of EGT × EPTp2 and EGT × EPTp3 are significantly negative at the 5% level. The absolute values of the coefficients were significantly smaller than EGT × EPTp1, and those difference are statistically significant at the 1% level. However, their coefficients’ absolute values and statistical significance were better than those of the last set of cross-products EGT × EPTp4.

In addition, we decompose EPThc into three variables according to the specificity of mentioning numerical control targets for pollutants: specific numerical control targets that mention specific pollutants or energy consumption per unit of GDP (EPTn1), mentioning environmental protection targets but not specific numerical control targets (EPTn2), and not actively mentioning environmental protection targets (EPTn3). The results are shown in column (3) of Table 4, and the coefficients of all three sets of interaction terms are negative. However, among them, the absolute value and statistical significance of the coefficient of EGT × EPTn1 are better than those of the latter two groups, and those difference are statistically significant at the 1% level. The above results show that the more specific the pollutant or numerical control target mentioned by local officials in the government work report involving environmental protection targets, the more pronounced the role of environmental protection targets in mitigating the negative environmental externalities of economic growth targets. Moreover, the more explicit implicit targets can enhance the coordination effect of policy targets.

More importantly, the sum of the coefficients between EGT × EPTp1 and EGT in column (2) of Table 9 is less than zero, as are the coefficients of EGT × EPTn1 and EGT in column (3). This shows that the mention of specific pollutants or numerical control targets in the government work report can effectively drive local officials to transform the hard constraints into self-constraints of environmental protection targets, reversing the negative environmental externalities of economic growth targets.

## 7. Conclusions

This paper investigates how local governments coordinate the relationship between economic growth and environmental protection targets and the resulting environmental governance performance. Results show that economic growth target pressure is significantly positively related to firm-level SO_2_ emission intensity and that the local government’s relaxation of environmental regulations is a potential impact mechanism. This indicates that the economic growth target has negative environmental externalities. Further, we find that the hard constraints of environmental protection targets by the central government partially alleviate the environmental externalities of the economic growth target, which is mainly realized by driving local governments to strengthen environmental regulation. Finally, we show that converting hard constraints of environmental protection targets to self-constraint by local government officials could reverse the negative environmental externality of economic growth target and truly realize the coordination of economic growth targets and environmental protection targets.

Our study adds to the growing literature on the tensions and trade-offs between economic development and environmental stewardship and provides empirical evidence of the environmental externality related to rapid economic development. Therefore, it has policy implications for balancing the relationship between economic development and environmental sustainability.

## Figures and Tables

**Table 1:** Sample composition.

**Panel A Year Distribution**			
**Year**	**# of Observations**	**% of Observations**	**Mean of SO_2_ Emission**
2003	23,086	7.16%	1.53
2004	26,102	8.10%	1.46
2005	25,747	7.99%	1.25
2006	30,188	9.37%	0.93
2007	40,330	12.52%	0.78
2008	36,246	11.25%	0.68
2009	36,111	11.21%	0.56
2011	40,282	12.50%	0.38
2012	37,622	11.68%	0.36
2013	26,498	8.22%	0.53
Total	322,212	100.00%	0.79
**Panel B Industry Distribution**			
**Industry Name**	**# of Observations**	**% of Observations**	**Mean of SO_2_ Emission**
Mining and Washing of Coal	9088	2.82%	0.35
Extraction of Petroleum and Natural Gas	171	0.05%	0.22
Mining of Ferrous Metal Ores	3907	1.21%	0.28
Mining of Non-ferrous Metal Ores	3195	0.99%	0.22
Mining and Processing of Nonmetal Ores	1982	0.62%	1.08
Other Mining	8	0.00%	0.02
Processing of Food from Agricultural Products	20,944	6.50%	0.27
Manufacture of Foods	11,004	3.42%	0.36
Manufacture of Beverage	8568	2.66%	0.45
Manufacture of Tobacco	568	0.18%	0.36
Manufacture of Textile	28,061	8.71%	0.66
Manufacture of Textile Wearing Apparel	4353	1.35%	0.30
Manufacture of Leather	5069	1.57%	0.15
Manufacture of Timbers	4629	1.44%	0.48
Manufacture of Furniture	1404	0.44%	0.11
Manufacture of Paper	15,660	4.86%	1.29
Printing, Reproduction of Recording Media	2014	0.63%	0.11
Manufacture of Articles for Culture, Education and Sport Activities	1535	0.48%	0.09
Processing of Petroleum, Coking, Processing of Nuclear Fuel	3806	1.18%	1.73
Manufacture of Chemical Raw Material and Chemical Products	38,717	12.02%	0.67
Manufacture of Medicines	12,912	4.01%	0.28
Manufacture of Chemical Fiber	1597	0.50%	0.38
Manufacture of Rubber	3861	1.20%	0.38
Manufacture of Plastic	8566	2.66%	0.51
Manufacture of Nonmetallic Mineral Products	40,137	12.46%	2.21
Manufacture and Processing of Ferrous Metals	10,440	3.24%	0.64
Manufacture & Processing of Non-ferrous Metals	7887	2.45%	0.59
Manufacture of Metal Products	13,048	4.05%	0.16
Manufacture of General Purpose Machinery	13,649	4.24%	0.16
Manufacture of Special Purpose Machinery	5531	1.72%	0.11
Manufacture of Transport Equipment	10,834	3.36%	0.09
Manufacture of Electrical Machinery and Equipment	9175	2.85%	0.07
Manufacture of Communication Equipment	8222	2.55%	0.04
Manufacture of Measuring Instrument	2030	0.63%	0.06
Manufacture of Artwork	3868	1.20%	0.13
Recycling and Disposal of Waste	439	0.14%	0.21
Production and Supply of Electric and Heat Power	4851	1.51%	7.98
Production and Distribution of Gas	142	0.04%	1.46
Production and Distribution of Water	340	0.11%	0.20
Total	322,212	100.00%	0.79

Notes: This panel A lists the year distribution of the main sample. The unit of SO_2_ emission is a kilogram, and the unit of gross industrial output is one thousand CNY; This panel B lists the industry distribution of the main sample. # represents the total number of observations.

**Table 2:** Summary statistics.

Variables	# of Observations	Mean	Std.	Min	p25	p50	p75	Max
Emission	322,212	0.786	2.560	0.000	0.000	0.060	0.413	24.460
EGT	322,212	0.840	0.367	0.000	1.000	1.000	1.000	1.000
EPThc	322,212	0.767	0.422	0.000	1.000	1.000	1.000	1.000
SIZE	322,212	17.990	1.648	14.740	16.780	17.840	19.070	22.290
ROA	322,212	0.106	0.156	0.000	0.015	0.047	0.122	0.774
LEV	322,212	0.546	0.260	0.023	0.359	0.556	0.733	1.539
AGE	322,212	2.284	0.758	0.000	1.792	2.303	2.708	4.111
SOE	322,212	0.156	0.363	0.000	0.000	0.000	0.000	1.000
PERGDP	322,212	1.149	0.883	−0.739	0.523	1.121	1.749	3.369
MKTIDX	322,212	7.556	1.744	4.170	6.230	7.500	8.810	11.390
ERS	322,212	0.386	0.166	0.126	0.250	0.386	0.464	0.991

Notes: # represents the total number of observations.

**Table 3:** Baseline regressions.

	Model (1)		Model (2)	
	(1)	(2)	(3)	(4)
EGT	0.048 ***	0.060 ***	0.151 ***	0.154 ***
	(2.68)	(3.32)	(4.51)	(4.56)
EGT × EPThc			−0.142 ***	−0.131 ***
			(−4.13)	(−3.73)
SIZE	−0.143 ***	−0.141 ***	−0.143 ***	−0.141 ***
	(−33.61)	(−33.26)	(−33.61)	(−33.27)
ROA	−1.011 ***	−0.980 ***	−1.007 ***	−0.977 ***
	(−36.18)	(−35.08)	(−35.99)	(−34.96)
LEV	0.140 ***	0.130 ***	0.139 ***	0.129 ***
	(5.42)	(5.04)	(5.40)	(5.02)
AGE	0.056 ***	0.054 ***	0.056 ***	0.054 ***
	(5.90)	(5.65)	(5.92)	(5.66)
SOE	0.068 ***	0.068 ***	0.069 ***	0.069 ***
	(3.42)	(3.40)	(3.47)	(3.45)
PERGDP		−0.554 ***		−0.532 ***
		(−9.18)		(−8.69)
MKTIDX		−0.100 ***		−0.102 ***
		(−9.25)		(−9.34)
ERS		−0.300 ***		−0.320 ***
		(−10.12)		(−10.85)
Constant	3.206 ***	4.685 ***	3.212 ***	4.683 ***
	(39.18)	(38.28)	(39.25)	(38.26)
Year FE	Yes	Yes	Yes	Yes
Ind FE	Yes	Yes	Yes	Yes
City FE	Yes	Yes	Yes	Yes
R-squared	0.259	0.260	0.259	0.260
Observations	322,212	322,212	322,212	322,212

Notes: The table reports the baseline regressions of Model (1) and Model (2). We include year, industry, and city fixed effects. Columns (1) and (3) control for the firm-level characteristics, and columns (2) and (4) further control for municipal and provincial characteristics. Variable definitions are in the Appendix A. Standard errors are clustered at the firm level and are reported in parentheses. *** indicate significance at the 1% levels, respectively.

**Table 4:** IV estimations.

	First-Stage	Second-Stage	First-Stage	Second-Stage
Dependent Variable	EGT	Emission	EGT	Emission
	(1)	(2)	(3)	(5)
$\hat{\mathrm{EGT}}$		4.806 ***		7.788 ***
		(7.10)		(8.37)
$\hat{\mathrm{EGT}}\times$ EPThc				−3.051 ***
				(−9.68)
EGTiv	0.108 ***		0.190 ***	
	(13.00)		(16.81)	
EGTiv × EPThc			−0.135 ***	
			(−14.24)	
SIZE	−0.001 ***	−0.137 ***	−0.001 ***	−0.137 ***
	(−2.94)	(−30.75)	(−3.24)	(−29.96)
ROA	0.039 ***	−1.170 ***	0.039 ***	−1.136 ***
	(14.81)	(−28.53)	(14.97)	(−26.72)
LEV	−0.004 **	0.149 ***	−0.004 **	0.141 ***
	(−2.47)	(5.51)	(−2.54)	(5.08)
AGE	0.000	0.052 ***	0.000	0.056 ***
	(0.65)	(5.24)	(0.56)	(5.43)
SOE	−0.001	0.073 ***	−0.001	0.094 ***
	(−0.80)	(3.53)	(−0.55)	(4.35)
PERGDP	−0.014 **	−0.539 ***	−0.012 **	−0.016
	(−2.37)	(−7.98)	(−2.05)	(−0.19)
MKTIDX	0.022 ***	−0.197 ***	0.022 ***	−0.244 ***
	(17.81)	(−10.66)	(17.80)	(−10.99)
ERS	0.048 ***	−0.559 ***	0.061 ***	−1.086 ***
	(10.90)	(−10.82)	(12.89)	(−11.62)
Year FE	Yes	Yes	Yes	Yes
Ind FE	Yes	Yes	Yes	Yes
City FE	Yes	Yes	Yes	Yes
Observations	322,212	322,212	322,212	322,212
Underidentification test	164.149		135.653	
Cragg–Donald F-statistic	352.804		143.934	
Kleibergen–Paap rk F-statistic	169.020		69.598	

Notes: The table presents the two-stage IV estimations of Model (1) and Model (2). We use the proportion of the top-down amplification of other city’s economic growth target in the same province as the instrumental variable EGTiv. In columns (1) and (3), the weak instrumental variable tests report both Cragg–Donald Wald F statistics and Kleibergen–Paap rk F-statistic. The underidentification test of instrumental variables adopts Kleibergen–Paap rk LM statistic. ** and *** indicate significance at the 5%, and 1% levels, respectively.

**Table 5:** Alternative measures of pollutants.

Dependent Variable	COD		IWG		IWW	
	(1)	(2)	(3)	(4)	(5)	(6)
EGT	0.241	0.939 ***	0.016 ***	0.034 ***	0.228 ***	0.208 ***
	(1.09)	(2.59)	(4.64)	(7.45)	(6.12)	(3.44)
EGT × EPThc		−1.000 ***		−0.031 ***		0.018
		(−2.84)		(−6.67)		(0.28)
Controls	Yes	Yes	Yes	Yes	Yes	Yes
Year FE	Yes	Yes	Yes	Yes	Yes	Yes
Ind FE	Yes	Yes	Yes	Yes	Yes	Yes
City FE	Yes	Yes	Yes	Yes	Yes	Yes
R-squared	0.147	0.147	0.320	0.320	0.219	0.262
Observations	384,861	384,861	320,523	320,523	388,452	388,449

Notes: The table reports the regressions of Model (1) and (2) using alternative measures of pollutants. Standard errors are clustered at the firm level and reported in parentheses. *** indicate significance at the 1% levels, respectively.

**Table 6:** Sample selection.

	Excluding Observations with Operating Income Less than 20 Million CNY		Excluding Observations if Variable Emission = 0		Excluding the Sample of 2013	
	(1)	(2)	(3)	(4)	(5)	(6)
EGT	0.014	0.104 ***	0.078 ***	0.149 ***	0.051 **	0.148 ***
	(0.84)	(3.15)	(3.37)	(3.46)	(2.56)	(4.38)
EGT × EPThc		−0.107 ***		−0.101 **		−0.144 ***
		(−3.19)		(−2.20)		(−4.10)
Controls	Yes	Yes	Yes	Yes	Yes	Yes
Year FE	Yes	Yes	Yes	Yes	Yes	Yes
Ind FE	Yes	Yes	Yes	Yes	Yes	Yes
City FE	Yes	Yes	Yes	Yes	Yes	Yes
R-squared	0.316	0.291	0.277	0.277	0.266	0.266
Observations	272,400	272,403	233,085	233,085	295,714	295,714

Notes: The table reports the regressions of Model (1) and (2) using subsample regression. Standard errors are clustered at the firm level and reported in parentheses. **, and *** indicate significance at the 5%, and 1% levels, respectively.

**Table 7:** Other robustness tests.

	Logarithm of SO_2_ Emissions		Emission Scaled by Income		Firm Fixed Effects	
Dependent Variable	LnSO_2_		Emission_Sale		Emission	
	(1)	(2)	(3)	(4)	(5)	(6)
EGT	0.100 ***	0.187 ***	0.055 ***	0.161 ***	0.027 *	0.147 ***
	(3.48)	(4.03)	(2.88)	(4.45)	(1.67)	(4.84)
EGT × EPThc		−0.120 **		−0.147 ***		−0.164 ***
		(−2.48)		(−3.92)		(−5.53)
TCP						
TCP_EPThc						
Controls	Yes	Yes	Yes	Yes	Yes	Yes
Year FE	Yes	Yes	Yes	Yes	Yes	Yes
Ind FE	Yes	Yes	Yes	Yes	Yes	Yes
City FE	Yes	Yes	Yes	Yes	Yes	Yes
R-squared	0.297	0.297	0.255	0.255	0.747	0.748
Observations	322,212	322,212	322,179	322,179	291,257	291,257

Notes: The table reports other robustness tests of Model (1) and (2). Standard errors are clustered at the firm level and reported in parentheses. *, **, and *** indicate significance at the 10%, 5%, and 1% levels, respectively.

**Table 8:** Underlying mechanism: environmental regulation.

**Panel A: Firm Level**									
	**Desulfurization Facilities**			**Desulfurization Capacity**			**Desulfurization Ratio**		
	**LnDnum**			**LnDcap**			**RemoveRate**		
	**(1)**	**(2)**		**(3)**	**(4)**		**(5)**		**(6)**
EGT	−0.004 *	−0.023 ***		−0.105 ***	−0.146 ***		−0.033 ***		−0.064 ***
	(−1.78)	(−5.55)		(−6.75)	(−7.07)		(−3.42)		(−7.54)
EGT × EPThc		0.030 ***			0.068 ***				0.045 ***
		(6.84)			(3.22)				(5.26)
SIZE	0.018 ***	0.018 ***		0.111 ***	0.111 ***		0.015 ***		0.015 ***
	(32.13)	(32.15)		(28.46)	(28.46)		(12.03)		(12.03)
ROA	−0.004	−0.005		−0.083 ***	−0.084 ***		−0.019		−0.020
	(−1.21)	(−1.39)		(−2.62)	(−2.67)		(−1.35)		(−1.42)
LEV	0.017 ***	0.017 ***		0.137 ***	0.137 ***		0.057 ***		0.057 ***
	(7.23)	(7.24)		(7.81)	(7.81)		(8.40)		(8.43)
AGE	0.004 ***	0.004 ***		0.015 **	0.015 **		0.005 **		0.004 **
	(4.31)	(4.26)		(2.29)	(2.27)		(2.16)		(2.14)
SOE	0.027 ***	0.027 ***		0.138 ***	0.138 ***		0.031 ***		0.030 ***
	(11.26)	(11.14)		(8.50)	(8.45)		(5.63)		(5.58)
PERGDP	−0.006	−0.011 *		0.133 ***	0.123 ***		0.081 ***		0.072 ***
	(−1.03)	(−1.74)		(3.69)	(3.38)		(3.34)		(2.96)
MKTIDX	0.008 ***	0.007 ***		0.002	0.001		−0.014 ***		−0.014 ***
	(5.90)	(5.75)		(0.18)	(0.13)		(−3.10)		(−3.03)
ERS	−0.006	0.002		−0.003	0.014		−0.176 ***		−0.169 ***
	(−1.05)	(0.34)		(−0.07)	(0.41)		(−12.32)		(−11.83)
Constant	−0.327 ***	−0.326 ***		−1.854 ***	−1.853 ***		−0.156 ***		−0.154 ***
	(−22.34)	(−22.31)		(−18.46)	(−18.45)		(−3.29)		(−3.26)
Year FE	Yes	Yes		Yes	Yes		Yes		Yes
Ind FE	Yes	Yes		Yes	Yes		Yes		Yes
City FE	Yes	Yes		Yes	Yes		Yes		Yes
R-squared	0.174	0.175		0.156	0.156		0.096		0.096
Observations	133,772	133,772		133,772	133,772		228,464		228,464
**Panel B: Industry Level**									
	**Model (1)**					**Model (2)**			
	**High Exits of Dirty** **Industries**		**Low Exits of Dirty** **Industries**			**High Exits of Dirty** **Industries**		**Low Exits of Dirty** **Industries**	
	**(1)**		**(2)**			**(3)**		**(4)**	
EGT	0.020		0.256 ***			0.096 **		0.251 ***	
	(1.04)		(5.89)			(2.56)		(3.46)	
EGT × EPThc						−0.103 ***		0.009	
						(−2.62)		(0.10)	
SIZE	−0.135 ***		−0.153 ***			−0.135 ***		−0.153 ***	
	(−30.33)		(−20.35)			(−30.33)		(−20.35)	
ROA	−1.020 ***		−0.897 ***			−1.017 ***		−0.897 ***	
	(−32.16)		(−19.59)			(−32.03)		(−19.60)	
LEV	0.111 ***		0.152 ***			0.110 ***		0.152 ***	
	(3.95)		(3.38)			(3.94)		(3.38)	
AGE	0.059 ***		0.035 **			0.059 ***		0.035 **	
	(5.85)		(2.03)			(5.86)		(2.03)	
SOE	0.032		0.118 ***			0.033		0.118 ***	
	(1.54)		(3.42)			(1.58)		(3.43)	
PERGDP	−0.507 ***		−0.531 ***			−0.488 ***		−0.532 ***	
	(−7.90)		(−4.85)			(−7.43)		(−4.82)	
MKTIDX	−0.083 ***		−0.103 ***			−0.083 ***		−0.103 ***	
	(−6.77)		(−4.57)			(−6.80)		(−4.54)	
ERS	−0.291 ***		−0.702 ***			−0.312 ***		−0.702 ***	
	(−8.99)		(−7.20)			(−9.73)		(−7.20)	
Constant	4.452 ***		4.803 ***			4.441 ***		4.801 ***	
	(32.20)		(22.48)			(32.10)		(22.44)	
Year FE	Yes		Yes			Yes		Yes	
Ind FE	Yes		Yes			Yes		Yes	
City FE	Yes		Yes			Yes		Yes	
R-squared	0.243		0.297			0.243		0.297	
Observations	219,967		100,757			219,967		100,757	

Notes: The table reports the underlying mechanism by which the coordination of multi-tasking policy targets affects pollution emissions. Standard errors are clustered at the firm level and reported in parentheses. *, **, and *** indicate significance at the 10%, 5%, and 1% levels, respectively.

**Table 9:** Reverse the positive trend of EGT and Emission.

	Self-Constraints of EPT	Mention of Specific Pollutants of EPT	Mention of Specific Numerical Targets of EPT
	(1)	(2)	(3)
EGT × EPTsc	−0.246 ***		
	(−5.77)		
EGT × EPTnsc	−0.068 *		
	(−1.91)		
EPTsc	0.164 ***		
	(5.35)		
EGT × EPTp1		−0.248 ***	
		(−5.81)	
EGT × EPTp2		−0.080 **	
		(−2.13)	
EGT × EPTp3		−0.105 **	
		(−1.97)	
EGT × EPTp4		−0.063	
		(−1.41)	
EPTp1		0.159 ***	
		(4.17)	
EPTp2		−0.016	
		(−0.46)	
EPTp3		0.086 *	
		(1.69)	
EGT × EPTn1			−0.175 ***
			(−4.60)
EGT × EPTn2			−0.055
			(−1.44)
EGT × EPTn3			−0.049
			(−1.12)
EPTn1			0.069 **
			(2.02)
EPTn2			0.021
			(0.59)
EGT	0.157 ***	0.163 ***	0.150 ***
	(4.63)	(4.77)	(4.48)
SIZE	−0.141 ***	−0.141 ***	−0.141 ***
	(−33.26)	(−33.27)	(−33.26)
ROA	−0.978 ***	−0.978 ***	−0.979 ***
	(−34.98)	(−35.01)	(−35.01)
LEV	0.130 ***	0.130 ***	0.129 ***
	(5.03)	(5.04)	(5.01)
AGE	0.054 ***	0.054 ***	0.054 ***
	(5.67)	(5.66)	(5.64)
SOE	0.069 ***	0.069 ***	0.068 ***
	(3.46)	(3.45)	(3.43)
PERGDP	−0.542 ***	−0.538 ***	−0.544 ***
	(−8.83)	(−8.76)	(−8.84)
MKTIDX	−0.098 ***	−0.098 ***	−0.103 ***
	(−9.05)	(−8.98)	(−9.42)
ERS	−0.291 ***	−0.288 ***	−0.310 ***
	(−9.67)	(−9.53)	(−10.41)
Constant	4.610 ***	4.601 ***	4.664 ***
	(37.66)	(37.35)	(37.74)
Year FE	Yes	Yes	Yes
Ind FE	Yes	Yes	Yes
City FE	Yes	Yes	Yes
R-squared	0.260	0.260	0.260
Observations	322,212	322,212	322,212

Notes: The table reports the results of whether the environmental protection targets’ self-restraint could reverse the economic growth target’s negative environmental externality trend. Standard errors are clustered at the firm level and reported in parentheses. *, **, and *** indicate significance at the 10%, 5%, and 1% levels, respectively.

## Data Availability

The data presented in this study are available on request from the corresponding author.

## Abbreviations

CIED: China Industrial Enterprise Database; CNY, Chinese yuan; EPB, Environmental Protection Bureau; ESR, China Environmental Survey and Reporting Database; EGT, economic growth targets; EPT, environmental protection targets.

## Appendix A. Variable Definitions

**Table A1:** The definitions of the main variables used in this study.

Variables	Definition
Emission variables	
Emission	The amount of total SO_2_ emission (in kilograms) scaled by total output (per thousand CNY), adjusted for inflation.
Emission^sale^	The amount of total SO_2_ emission (in kilograms) scaled by operating income (per thousand CNY), adjusted for inflation.
LnSO_2_	the logarithm of SO_2_ emissions (in kilograms) at the firm level
Independent variables	
EGT	Economic growth target pressure, a dummy variable taking the value of one if the GDP growth target set by the municipal government in a given year is higher than the provincial GDP growth target and 0 otherwise
EPThc	The environmental policy target’s hard constraint variable, assigning a value of one to the sample observations from 2007 to 2013, with years before 2007 assigned a value of zero
EPTsc	If local officials voluntarily disclose the emission reduction numerical targets of major pollutants in the government work report after 2007, the EPTsc value is 1; otherwise, it is 0.
EPTnsc	if the local officials did not disclose numerical targets of major pollutants in the government work report after 2007, EPTnsc takes the value 1; otherwise, it is 0.
EPTp1	Mentioning the specific pollutant (sulfur dioxide or nitrogen oxides) control target in government work reports under the hard constraints of environmental protection targets
EPTp2	Mentioning the energy consumption per unit of GDP control target only in government work reports under the hard constraints of environmental protection targets
EPTp3	Mentioning the completion of the higher-level government target only in government work reports under the hard constraints of environmental protection targets
EPTp4	Facing hard constraints from the higher government but not actively mentioning the environmental protection target
EPTn1	Mentioning specific numerical control targets of specific pollutants or energy consumption per unit of GDP in government work reports under the hard constraints of environmental protection targets
EPTn2	Mentioning environmental protection targets but not specific numerical control targets in government work reports under the hard constraints of environmental protection targets
EPTn3	Not actively mentioning environmental protection targets under the hard constraints of environmental protection targets
Instrumental variable	
EGTiv	The proportion of the top-down amplification of another city’s economic growth target in the same province as the instrumental variable
Mechanism variables	
DirtyExit	A dummy variable, taking a value of one if the number of heavy polluting firms exiting the city in a given year is higher than the city-level median and zero otherwise
Firm-level control variables	
SIZE	The natural logarithm of total assets at the firm level
ROA	Net income scaled by total assets at the firm level
LEV	Total debt scaled by total assets at the firm level
AGE	The natural logarithm of the age of the firm
SOE	An indicator variable set to one if the firm owner is a state-owned firm and zero otherwise.
Municipal and provincial control variables	
MKTIDX	The provincial-level marketization degree constructed by the existing literature [34]
ERS	Provincial environmental regulation index following the existing literature [35], investment in industrial pollution control divided by total environmental pollution emissions
PERGDP	GDP per capita at the prefecture-level city

## References

[1] Lyu C., Wang K., Zhang F., Zhang X. (2018). GDP management to meet or beat growth targets. J. Account. Econ..

[2] Liu D., Xu C., Yu Y., Rong K., Zhang J. (2020). Economic growth target, distortion of public expenditure and business cycle in China. China Econ. Rev..

[3] Li H., Zhou L.A. (2005). Political turnover and economic performance: The incentive role of personnel control in China. J. Public Econ..

[4] Yao Y., Zhang M.Y. (2015). Subnational leaders and economic growth: Evidence from Chinese cities. J. Econ. Growth.

[5] Li X., Liu C., Weng X., Zhou L.A. (2019). Target setting in tournaments: Theory and evidence from China. Econ. J..

[6] Konisky D.M., Woods N.D. (2012). Environmental free riding in state water pollution enforcement. State Politics Policy Q..

[7] Grooms K.K. (2015). Enforcing the Clean Water Act: The effect of state-level corruption on compliance. J. Environ. Econ. Manag..

[8] Piotroski J.D., Wong T.J. (2012). Institutions and Information Environment of Chinese Listed Firms. Capitalizing China.

[9] Kim I., Wan H., Wang B., Yang T. (2019). Institutional investors and corporate environmental, social, and governance policies: Evidence from toxics release data. Manag. Sci..

[10] Liu Z., Shen H., Welker M., Zhang N., Zhao Y. (2021). Gone with the wind: An externality of earnings pressure. J. Account. Econ..

[11] Yu Y., Liu D., Gong Y. (2019). Target of local economic growth and total factor productivity. Manag. World.

[12] Shen F., Liu B., Luo F., Wu C., Chen H., Wei W. (2021). The effect of economic growth target constraints on green technology innovation. J. Environ. Manag..

[13] Locke E. (2000). Motivation, cognition, and action: An analysis of studies of task goals and knowledge. Appl. Psychol..

[14] Holmstrom B., Milgrom P. (1991). Multitask principal-agent analyses: Incentive contracts, asset ownership, and job design. J. Law Econ. Organ..

[15] Blanchard O., Shleifer A. (2001). Federalism with and without political centralization: China versus Russia. IMF Staff. Pap..

[16] Tsui K.Y., Wang Y. (2004). Between separate stoves and a single menu: Fiscal decentralization in China. China Q..

[17] Jin H., Qian Y., Weingast B. (2005). Regional decentralization and fiscal incentives: Federalism, Chinese style. J. Public Econ..

[18] Xu C. (2011). The fundamental institutions of China’s reforms and development. J. Econ. Lit..

[19] Liu Y., Hao Y., Gao Y. (2017). The environmental consequences of domestic and foreign investment: Evidence from China. Energy Policy.

[20] Greenstone M., List J.A., Syverson C. (2012). The Effects of Environmental Regulation on the Competitiveness of US Manufacturing (No. w18392).

[21] Xu Y. (2011). Improvements in the Operation of SO_2_ Scrubbers in China’s Coal Power Plants. Environ. Sci. Technol..

[22] Karplus V.J., Wu M. (2019). Crackdowns in Hierarchies: Evidence from China’s Environmental Inspections. MIT Sloan Research Paper No. 5700-19.

[23] Tao F., Zhao J., Zhou H. (2021). Does environmental regulation improve the quantity and quality of green innovation: Evidence from the target responsibility system of environmental protection. China Ind. Econ..

[24] Chen Y.J., Li P., Lu Y. (2018). Career concerns and multitasking local bureaucrats: Evidence of a target-based performance evaluation system in China. J. Dev. Econ..

[25] He G., Wang S., Zhang B. (2020). Watering down environmental regulation in China. Q. J. Econ..

[26] Brandt L., Van Biesebroeck J., Zhang Y. (2012). Creative accounting or creative destruction? Firm-level productivity growth in Chinese manufacturing. J. Dev. Econ..

[27] Deng Y., Wu Y., Xu H. (2019). Political turnover and firm pollution discharges: An empirical study. China Econ. Rev..

[28] Chen Y., Li H., Zhou L.A. (2005). Relative performance evaluation and the turnover of provincial leaders in China. Econ. Lett..

[29] Yu J., Zhou L.A., Zhu G. (2016). Strategic interaction in political competition: Evidence from spatial effects across Chinese cities. Reg. Sci. Urban Econ..

[30] Li F., Wang Z., Huang L. (2021). Economic growth target and environmental regulation intensity: Evidence from 284 cities in China. Environ. Sci. Pollut. Res..

[31] Yu Y.Z., Sun P.B., Xuan Y. (2020). Do Constraints on Local Governments’ Environmental Targets Affect Industrial Transformation and Upgrading. J. Econ. Res..

[32] Dewatripont M., Jewitt I., Tirole J. (1999). The economics of career concerns, part II: Application to missions and accountability of government agencies. Rev. Econ. Stud..

[33] Alesina A., Tabellini G. (2008). Bureaucrats or politicians? Part II: Multiple policy tasks. J. Public Econ..

[34] Fan G., Wang X., Yu J. (2017). Marketization Index of China’s Provinces: NERI Report 2016.

[35] Wu H., Hao Y., Ren S. (2020). How do environmental regulation and environmental decentralization affect green total factor energy efficiency: Evidence from China. Energy Econ..

